# Statistical testing and spatio-temporal evolution characteristics of the coupling coordination between green agricultural production efficiency and food security

**DOI:** 10.1371/journal.pone.0328787

**Published:** 2025-07-23

**Authors:** Tianao Li, Yue Hu

**Affiliations:** School of Marxism, Dalian University of Technology, Dalian, China; National Cheng Kung University, TAIWAN

## Abstract

Under the requirements of developing new quality productivity, balancing green agricultural development with food security has become crucial for sustainable development, particularly in regions facing environmental constraints. This study investigates the coupling coordination relationship between agricultural green development efficiency and food security in China’s agricultural system. Using panel data from 31 Chinese provinces (2010–2022), the study employs a comprehensive analytical framework combining the super-efficiency SBM-DEA model for efficiency measurement, the entropy TOPSIS method for food security evaluation, coupling coordination degree analysis, and multi-dimensional spatial analysis including regional difference decomposition, spatial correlation analysis, and kernel density estimation. The results reveal three distinct development phases: stable development (2010–2016, coordination degree 0.49–0.52), rapid improvement (2017–2018, peaking at 0.60), and stable adjustment (2019–2022, stabilizing around 0.54). Spatially, three high-value clusters have formed around Beijing-Tianjin-Hebei (average coordination degree 0.68), the Yangtze River Delta (0.76), and Heilongjiang (0.81). Significant regional disparities persist, with eastern regions maintaining stable high-level development (average coordination degree >0.65) and western regions generally remaining below 0.50. Despite facing structural development constraints, Western regions possess latent comparative advantages in ecological agriculture. Gini coefficient decomposition, Moran’s index analysis, and kernel density estimation collectively reveal that between-region differences dominate inequality sources (36.19%–49.99%), with strengthening spatial agglomeration effects creating path-dependent regional stratification. The findings suggest that achieving coordinated development requires differentiated regional strategies: technology transfer leadership for high-coordination regions, sustainable intensification support for transitional regions, and comprehensive structural interventions for low-coordination areas. These insights provide practical guidance for developing agricultural new quality productivity while ensuring food security.

## 1 Introduction

Severe challenges such as global climate change, resource constraints, and environmental pollution are driving agriculture toward green and sustainable transformation [1]. The Intergovernmental Panel on Climate Change (IPCC) reports that agricultural and land-use activities contributed 23% of net anthropogenic greenhouse gas emissions during 2007–2016. Meanwhile, inefficient use of agricultural inputs such as fertilizers in intensive agricultural production has caused serious non-point source pollution. The United Nations predicts that the global population will reach 10 billion by 2050, requiring global agricultural production to increase by 50% from 2010 levels to meet demand [2]. Against the background of increasingly tight resources, China faces even more prominent challenges: feeding 22% of the world’s population with only 7% of global arable land. To address this challenge, the government has proposed “dual carbon” goals and clearly stated the construction of a modernized green agricultural system in the “14th Five-Year Plan” [3]. However, agricultural green transformation is a gradual process, and its development efficiency directly affects both the speed and quality of transformation. Inefficient transformation not only exacerbates resource waste but may also impair food security due to rising production costs affecting food supply capacity. Therefore, scientifically assessing current agricultural green development efficiency and exploring its coupling mechanism with food security has important theoretical value and practical significance for formulating more precise and effective policies.

Currently, academia has achieved rich research results in the fields of agricultural green development efficiency and food security. Agricultural green development efficiency focuses on maximizing economic benefits while minimizing resource input and ecological damage [4]. From the perspective of evaluation systems, research generally adopts an input-output framework, using labor, capital, and energy as input indicators, while treating total agricultural output value and environmental pollution indicators as desirable and undesirable outputs, respectively [5]. Assessment methods have undergone significant evolution: from early use of DEA methods for basic efficiency calculation [6], to introduction of EBM models and ML indices with environmental constraints [7,8], and then to MinDS super-efficiency-MetaFrontier-Malmquist models capable of capturing regional heterogeneity [9]. The methodological system has been continuously improved, gradually expanding from traditional production efficiency calculation to comprehensive assessment under environmental constraints.

Regarding influencing factors, human capital investment has been proven to significantly promote agricultural green growth [10], while improvements in rural residents’ income levels play an important role in enhancing agricultural green total factor productivity [11]. The effective combination of financial support and environmental regulation is also considered an important means to promote agricultural green development. Under the research framework of agricultural sustainable development, academia also highly values food security research. Food security research has shifted from traditional single-dimensional evaluation to multi-dimensional comprehensive assessment. Research shows that food security evaluation systems should comprehensively consider multiple aspects such as production scale, per capita grain possession, infrastructure construction, and ecological sustainability [12].

Researchers use methods such as the Delphi method and entropy method to analyze the weighting of these indicators [13], where sustainable utilization of irrigation water conservancy and farmland resources [14] and completeness of transportation infrastructure [15] have been proven to be important foundations for ensuring food security. Given their close connection, scholars have begun to focus on their interaction mechanisms. Research has found that there exists an obvious two-way interaction between them: environmental regulation and green agricultural policies, while improving agricultural green development efficiency, may increase production costs and affect agricultural product supply capacity [16]; while technological innovation, policy optimization, and market regulation mechanisms may promote their coordinated development [17].

However, existing research has several limitations. First, theoretical frameworks require further development, as current studies lack systematic theoretical frameworks to explain the underlying interaction mechanisms and coordination dynamics between agricultural green development and food security. Most research treats these interactions as empirical associations without sufficient theoretical grounding to understand how different systems can achieve effective coordination. Second, research methods can be further improved, as current research is mainly based on static correlation analysis and needs to deepen discussion on the spatiotemporal dynamic laws of their coordinated development and its statistical significance. Third, research on spatial spillover effects can be further deepened, especially regarding interaction mechanisms and spatial transmission effects under different regional development phases. Fourth, regional differentiated policy research can be further strengthened, especially research on policy design targeting different regional characteristics and development stages. The expansion of these research directions will help to more comprehensively understand the relationship between agricultural green development and food security.

Based on this, this study focuses on the coupling coordination relationship between agricultural green development efficiency and food security, drawing upon coordination theory to construct a comprehensive analytical framework that helps explain interaction mechanisms through dependency management and coordination processes. The study constructs an agricultural green development efficiency evaluation system including resource utilization efficiency, environmental impact, economic benefits, and technological progress, as well as a food security evaluation framework covering supply guarantee, quality safety, and sustainability. Using super-efficiency SBM-DEA model, coupling coordination degree model and spatial econometric methods, this study systematically measures and analyzes the agricultural green development and food security levels of 31 Chinese provinces from 2010–2022, revealing their spatial heterogeneity and dynamic evolution characteristics, and exploring policy paths to promote their coordinated development. The innovation of this study is mainly reflected in three dimensions: theoretical framework, research methods, and practical application. In terms of theoretical framework, it establishes a systematic theoretical foundation by applying coordination theory concepts, constructing a unified analysis framework that helps explain why and how agricultural green development efficiency and food security interact through dependency management and coordination mechanisms. In terms of research methods, it innovatively introduces spatial econometric methods and statistical significance testing, constructing a multi-dimensional analytical system to systematically examine regional heterogeneity, spatial spillover effects, and temporal evolution patterns with theoretical justification for methodological choices. In terms of practical application, it provides theoretically-grounded differentiated agricultural development strategies and policy recommendations targeting different regional characteristics, offering practical policy guidance for promoting coordinated advancement of agricultural green development and food security.

The structure of the remaining parts of this paper is as follows: Sect 2 introduces the research methods, including main analytical tools such as the super-efficiency SBM-DEA model, entropy TOPSIS method, and coupling coordination degree model; Sect 3 conducts empirical analysis, first explaining data sources and variable selection, then carrying out systematic analysis from dimensions such as agricultural green development level, food security status, coupling coordination degree, regional differences, and spatial correlation; Sect 4 summarizes research findings based on empirical analysis results and proposes differentiated policy recommendations targeting different regional characteristics.

## 2 Research methodology

### 2.1 Theoretical framework and research design

This study constructs a systematic analytical framework based on coordination theory to evaluate the coupling coordination relationship and spatial distribution characteristics between China’s agricultural green development efficiency and food security. The framework applies coordination theory concepts through a comprehensive methodological approach that captures both static coordination states and dynamic evolution processes.

Coordination theory, as developed by Malone and Crowston (1994), focuses on managing dependencies between activities to achieve collective goals. The coupling coordination relationship between agricultural green development efficiency and food security vividly embodies the core ideas of coordination theory. In agricultural systems, coordination between green development efficiency and food security involves managing three critical dependencies: resource dependency (shared use of land, water, and inputs), temporal dependency (balancing short-term productivity with long-term sustainability), and outcome dependency (ensuring environmental improvements support rather than compromise food security). Effective coordination requires mechanisms that align incentives, reduce conflicts, and enable adaptive management across these dependencies. From this theoretical perspective, we identify four key coordination mechanisms: resource optimization (efficient use of shared resources to benefit both systems), environmental constraint management (addressing environmental limits that affect both systems), innovation diffusion (spreading beneficial practices across regions), and policy coordination (institutional alignment of green development and food security objectives).

**Fig 1:**
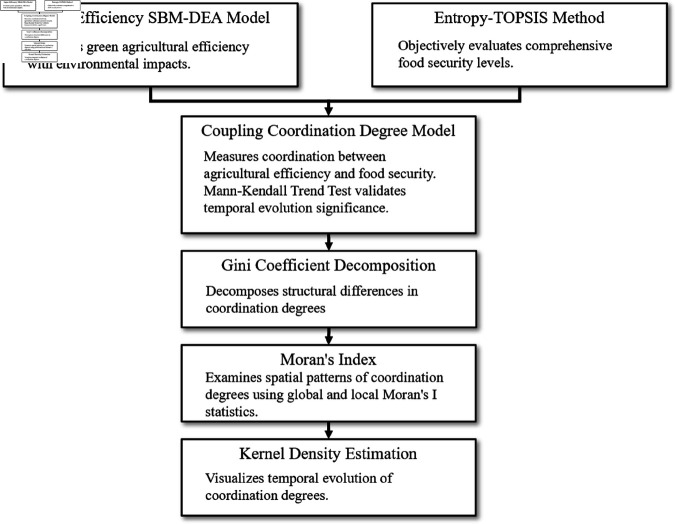
Research framework of this paper. The framework illustrates the systematic analysis approach combining multiple quantitative methods for evaluating coupling coordination between agricultural green development efficiency and food security.

The analytical framework translates these theoretical concepts into measurable constructs through six interconnected methodological modules that form a complete analytical chain. Beginning with system efficiency measurement, the super-efficiency SBM-DEA model captures how effectively agricultural systems manage resource dependencies by converting inputs into desired outputs while minimizing environmental impacts. Simultaneously, the entropy TOPSIS method captures the multi-dimensional nature of food security as conceptualized in coordination theory, recognizing that security emerges from coordinated management of multiple dependencies including production capacity, stability, accessibility, and sustainability. These two foundational measurements are then integrated through the coupling coordination degree model, which directly quantifies how well the two subsystems manage their dependencies and achieve mutually beneficial outcomes rather than conflicts, providing the core metric for understanding coordination dynamics. To understand the spatial and temporal patterns of this coordination, the analysis employs Gini coefficient decomposition to reveal regional coordination variations and their underlying sources, spatial autocorrelation analysis through Moran’s index to examine whether coordination improvements create spillover effects across neighboring regions, and kernel density estimation to capture how coordination patterns evolve over time and whether regions experience coordination transitions. Together, these methodological modules enable comprehensive examination of coordination mechanisms from individual system efficiency through regional coordination patterns to national spatiotemporal evolution dynamics. Fig 1 presents the overall research framework, illustrating the relationships between methodological modules and their integration into a comprehensive analytical system.

### 2.2 Super-efficiency SBM-DEA model

To address the problem that traditional DEA models cannot handle slack variables, Tone [18] proposed the SBM model. Since CCR and BCC models mainly focus on orientation analysis and proportion analysis, they cannot fully measure all slack variables, leading to inaccurate evaluation of decision-making units’ efficiency. The SBM model remedies this shortcoming of traditional DEA models, enabling precise calculation of redundant inputs and insufficient outputs, making efficiency evaluation results more accurate and reliable [19]. As scholars deepened their research on efficiency problems and DEA models, they found that traditional DEA models and SBM models still had limitations when dealing with multiple decision-making units in efficient states (i.e., scale efficiency equals 1). To further judge and compare efficiency differences among these efficient decision-making units, Tone [20] proposed the super-efficiency SBM-DEA model. The characteristic of this model is that the calculated efficiency values can be greater than or equal to 1, with higher efficiency values indicating more optimized input-output ratios of decision-making units, which helps conduct deeper analysis and comparison among different decision-making units in efficient states. This study adopts the super-efficiency SBM-DEA model to measure the agricultural green development efficiency of China’s 31 provincial-level administrative regions.

Input indicators encompass energy consumption (agricultural diesel, rural electricity use), labor input (rural population, labor force level), capital input (total agricultural machinery power, sown area), and resource input (pesticides, fertilizers, plastic film, etc.). Desirable outputs include the number of certified green food products, the number of certified units, and the standardized production base area, while undesirable outputs cover agricultural COD, carbon emissions, and ammonia nitrogen emissions.

Suppose there are *n* decision-making units that need to evaluate agricultural green development efficiency, denoted as ${\text{DMU}}_{j}(j=1,2,\ldots ,n)$, where each DMU represents a provincial-level administrative region in China. Each decision unit has *m* inputs, denoted as $x_{i}(i=1,2,\ldots ,m)$, *q*_1_ desirable outputs, denoted as $y_{r}(r=1,2,\ldots ,q_{1})$, and *q*_2_ undesirable outputs, denoted as $b_{t}(t=1,2,\ldots ,q_{2})$.

$$\;\min\;\rho =\frac{1+\frac{1}{m}\sum_{i=1}^{m}\frac{s_{i}^{-}}{x_{ik}}}{1-\frac{1}{q_{1}+q_{2}}\left(\sum_{r=1}^{q_{1}}\frac{s_{r}^{+}}{y_{ik}}+\sum_{t=1}^{q_{2}}\frac{s_{t}^{b-}}{b_{rk}}\right)},$$ (1)

$$\text{s.t.}\;\;\sum_{j=1,j\neq k}^{n}\lambda_{j}x_{ij}-s_{i}^{-}\;\leq \;x_{ik},\;i=1,2,\ldots ,m$$ (2)

$$\sum_{j=1,j\neq k}^{n}\lambda_{j}y_{rj}+s_{r}^{-}\geq y_{rk},$$ (3)

$$\sum_{j=1,j\neq k}^{n}\lambda_{j}b_{tj}-s_{t}^{b-}\leq b_{tk},$$ (4)

$$1-\frac{1}{q_{1}+q_{2}}\left(\sum_{r=1}^{q_{1}}\frac{s_{r}^{+}}{y_{ik}}+\sum_{t=1}^{q_{2}}\frac{s_{t}^{b-}}{b_{rk}}\right)>0,$$ (5)

Where λ≥0, $s^{-}\geq 0$, $s^{+}\geq 0$; i=1,2,…,q; j=1,2,…,n(j≠k).

### 2.3 Entropy TOPSIS method

The entropy TOPSIS method has clear advantages in objective weighting and multi-dimensional ranking [21]. Therefore, this study employs this method to comprehensively measure the food security evaluation indicator system constructed for China. First, we use the entropy method to standardize the evaluation indicator data *x*_*ij*_ for year *i* and indicator *j* and calculate their corresponding weights. Furthermore, after obtaining the weights, we calculate the weighted matrix, Euclidean distance, and comprehensive scores through the TOPSIS method.

#### 2.3.1 Data standardization.

For positive indicators:

$$x_{ij}^{\prime }=\frac{x_{ij}-\min(x_{j})}{\max(x_{j})-\min(x_{j})},$$ (6)

For negative indicators:

$$x_{ij}^{\prime }=\frac{\max(x_{j})-x_{ij}}{\max(x_{j})-\min(x_{j})},$$ (7)

#### 2.3.2 Determining weights using entropy method.

1) Calculate the proportion *P* of indicator *j* in year *i*:

$$P_{ij}=\frac{x_{ij}^{\prime }}{\sum_{i}x_{ij}^{\prime }},$$ (8)

where *P*_*ij*_ represents the relative proportion of standardized value $x_{ij}^{\prime }$ for indicator *j* in year *i* among all years.

2) Calculate the information entropy of indicator *j*:

$$e_{j}=-k\sum_{i}P_{ij}\ln(P_{ij}),$$ (9)

where *e*_*j*_ denotes the information entropy of indicator *j*, $k=\frac{1}{\ln(n)}$ is the normalization constant with *n* being the number of years, and *k* > 0, ensuring $e_{j}\;\geq \;0$. Higher entropy values indicate more uniform distribution across years.

3) Calculate the redundancy degree of information entropy for indicator *j*:

$$d_{j}=1-e_{j},$$ (10)

where *d*_*j*_ represents the information redundancy degree, measuring the variation level of indicator *j*. Higher *d*_*j*_ values indicate greater variation and thus higher information content.

4) Calculate the weight of indicator *j*:

$$\omega_{j}=\frac{d_{j}}{\sum_{j}d_{j}},$$ (11)

where $\omega_{j}$ is the normalized weight of indicator *j*, ensuring $\sum_{j}\omega_{j}=1$. Indicators with higher variation receive larger weights in the comprehensive evaluation.

#### 2.3.3 Calculating comprehensive scores using TOPSIS method.

1) Construct a weighted matrix:

$$X_{ij}=x_{ij}^{\prime }\times \omega_{j},$$ (12)

where *X*_*ij*_ represents the weighted standardized value for year *i* and indicator *j*, combining the standardized data $x_{ij}^{\prime }$ with the entropy-derived weight $\omega_{j}$.

2) Calculate Euclidean distance from ideal solutions:

$${D_{i}}^{+}=\sqrt{\sum_{j}(X_{ij}-{X_{j}}^{*+}{)}^{2}},$$ (13)

$${D_{i}}^{-}=\sqrt{\sum_{j}(X_{ij}-{X_{j}}^{*-}{)}^{2}},$$ (14)

where ${D_{i}}^{+}$ denotes the Euclidean distance from year *i* to the positive ideal solution ${X_{j}}^{*+}=\max_{i}(X_{ij})$ for each indicator *j*, and ${D_{i}}^{-}$ represents the distance to the negative ideal solution ${X_{j}}^{*-}=\min_{i}(X_{ij})$. Smaller ${D_{i}}^{+}$ and larger ${D_{i}}^{-}$ indicate better performance.

3) Calculate final comprehensive score:

$$C_{i}=\frac{D_{i}^{-}}{D_{i}^{+}+D_{i}^{-}},$$ (15)

where *C*_*i*_ is the comprehensive score for year *i*, ranging from 0 to 1. Higher *C*_*i*_ values indicate closer proximity to the ideal solution and better food security performance.

This comprehensive method combines the objectivity of entropy weighting with the systematic comparison capabilities of TOPSIS, providing a robust framework for evaluating food security levels. The entropy method helps eliminate subjective bias in weight assignment, while TOPSIS enables effective comparison of multiple alternatives by considering both positive and negative ideal solutions simultaneously.

### 2.4 Coupling coordination degree model

Building on the theoretical framework established above, the coupling coordination degree model employed in this study applies concepts from coordination theory to measure the interaction intensity and coordinated development level between China’s agricultural green development efficiency and food security systems. The coupling coordination degree reflects the extent to which multiple systems harmonize and align with each other based on their coupling relationship, providing a quantitative measure of how well the two subsystems manage their dependencies and achieve mutually beneficial outcomes rather than conflicts.

The coupling coefficient (C) captures the intensity of interaction between the two subsystems, reflecting the strength of dependencies identified in coordination theory. The coordination degree (D) measures the extent to which these interactions produce mutually beneficial outcomes rather than conflicts, embodying the concept of effective dependency management central to coordination theory. The specific formulas are as follows:

$$C=\sqrt{\frac{f(x)\times g(y)}{{[(f(x)+g(y))/2]}^{2}}},$$ (16)

T=a·f(x)+b·g(y), (17)

$$D=\sqrt{C\times T},$$ (18)

where *f*(*x*) represents the comprehensive score of agricultural green development efficiency, *g*(*y*) denotes the food security level score, *C* is the coupling coefficient measuring interaction intensity, *T* represents the comprehensive coordination index with weight coefficients a=b=0.5, and *D* is the final coupling coordination degree. The value range of *D* is [0,1], with higher values indicating better coordination.

The mathematical formulation reflects coordination theory principles: the multiplicative form f(x)×g(y) captures system interdependence, the normalization process ensures coordination assessment relative to each subsystem’s development level, and the geometric mean formulation recognizes that sustainable coordination requires balance rather than dominance of either subsystem.

This theoretical grounding provides a conceptual framework for interpreting empirical results and understanding why certain regions achieve higher coordination levels while others remain in low-coordination states.

### 2.5 Mann-Kendall trend test

To establish the statistical significance of temporal evolution patterns in coupling coordination between agricultural green development efficiency and food security, this research adopts the Mann-Kendall trend analysis. The Mann-Kendall approach represents a distribution-free statistical technique proposed by Mann [22]. This methodology requires no assumptions regarding data distribution normality and demonstrates robustness against measurement uncertainties and anomalous observations, making it particularly suitable for examining monotonic trends in time series data [23,24].

#### 2.5.1 Fundamental testing framework.

The Mann-Kendall trend analysis considers an independent temporal sequence $x_{1},x_{2},x_{3},\ldots ,x_{n}$. The primary test statistic *S* is determined through:

$$S=\sum_{i=1}^{n-1}\sum_{j=i+1}^{n}\text{sign}(x_{j}-x_{i}),$$ (19)

wherein the sign operator is specified as:

$$\text{sign}(x_{j}-x_{i})=\left\{ \begin{array}{cc} +1, & \text{if }x_{j}-x_{i}>0\\ 0, & \text{if }x_{j}-x_{i}=0\\ -1, & \text{if }x_{j}-x_{i}<0 \end{array}\right.$$ (20)

For temporal sequences where n≥10, the normalized test statistic *Z* is derived as:

$$Z=\left\{ \begin{array}{cc} \frac{S-1}{\sqrt{\text{Var}(S)}}, & \text{if }S>0\\ 0, & \text{if }S=0\\ \frac{S+1}{\sqrt{\text{Var}(S)}}, & \text{if }S<0 \end{array}\right.$$ (21)

The variance calculation for *S* follows:

$$\text{Var}(S)=\frac{n(n-1)(2n+5)-\sum_{i=1}^{m}t_{i}(t_{i}-1)(2t_{i}+5)}{18},$$ (22)

where *m* denotes the number of tied value groups and *t*_*i*_ represents the count of observations within the *i*-th tied group.

Under the significance threshold α=0.05, when |Z|≥1.96, the temporal pattern achieves statistical significance. Positive *Z* values signify upward trends, while negative values indicate downward patterns.

#### 2.5.2 Change point detection via UF and UB series.

For detecting structural discontinuities within the temporal sequence, this study implements the Mann-Kendall mutation detection utilizing UF (forward sequence) and UB (backward sequence) statistics. The UF statistical series emerges from sequential application of Mann-Kendall testing throughout the progressive temporal data:

$$UF_{k}=\frac{S_{k}-E(S_{k})}{\sqrt{\text{Var}(S_{k})}},\;k=2,3,\ldots ,n$$ (23)

The UB statistical series derives from time-reversed sequence analysis:

$$UB_{k}=-UF_{n+1-k}^{\prime },\;k=n,n-1,\ldots ,1$$ (24)

where $UF_{k}^{\prime }$ denotes the UF statistic computed from the time-reversed series.

When UF and UB trajectories intersect within the critical confidence boundaries (±1.96 at α=0.05), the intersection location signifies a statistically significant change point. This analytical approach facilitates precise identification of temporal moments experiencing structural transitions, thereby providing empirical support for the theorized developmental phases in coupling coordination evolution.

### 2.6 Gini coefficient and its decomposition

This study adopts the Gini coefficient decomposition framework [21,25], which decomposes overall inequality into within-region differences *G*_*w*_, between-region gaps *G*_*nb*_, and transvariation intensity *G*_*t*_, expressed as $G\;=\;G_{w}\;+\;G_{nb}\;+\;G_{t}$. The calculation formulas are:

$$G=\frac{\sum_{j=1}^{k}\sum_{h=1}^{k}\sum_{i=1}^{n_{j}}\sum_{r=1}^{n_{h}}\left|y_{ji}-y_{hr}\right|}{2\mu n^{2}},$$ (25)

$$G_{w}=\sum_{j=1}^{k}G_{jj}p_{j}s_{j},$$ (26)

$$G_{nb}=\sum_{j=2}^{k}\sum_{h=1}^{j-1}G_{jh}(p_{j}s_{h}+p_{h}s_{j})D_{jh},$$ (27)

$$G_{t}=\sum_{j=2}^{k}\sum_{h=1}^{j-1}G_{jh}(p_{j}s_{h}+p_{h}s_{j})(1-D_{jh}),$$ (28)

where *k* represents the total number of regions; *j* and *h* are region identifiers; *n* represents the number of provinces in the study area, with *n*_*j*_ and *n*_*h*_ being the number of provinces within regions *j* and *h*; *y*_*ji*_ and *y*_*hr*_ represent the agricultural green development level measurements for provinces in regions *j* and *h*; μ represents the arithmetic mean of the full sample measurements; $p_{j},s_{j}$ represent the population weight and income share of region *j*; *D*_*jh*_ represents the relative economic distance between regions *j* and *h*.

This decomposition method provides a comprehensive framework for understanding regional inequality, breaking down overall disparities into distinct components that reflect different aspects of spatial variation. The within-region component *G*_*w*_ captures local disparities, the between-region component *G*_*nb*_ reflects systematic regional gaps, and the transvariation intensity *G*_*t*_ measures the overlap between distributions of different regions.

### 2.7 Moran’s index

Moran’s Index measures spatial autocorrelation, including both global and local Moran’s Index [26]. In this study, we use the global Moran’s Index to examine whether there are significant clustering or dispersion trends in the coupling coordination degree among provinces. The local Moran’s Index helps identify four typical spatial association patterns, providing a basis for formulating differentiated regional policies.

#### 2.7.1 Global Moran’s index.

$$I=\frac{\sum_{i=1}^{n}\sum_{j=1}^{n}W_{ij}(D_{i}-\overset{―}{D})(D_{j}-\overset{―}{D})}{S^{2}\sum_{i=1}^{n}\sum_{j=1}^{n}W_{ij}},$$ (29)

#### 2.7.2 Local Moran’s index.

$$I_{i}=\frac{\left(D_{i}-\overset{―}{D}\right)}{S^{2}}\sum_{i\neq j}^{n}W_{ij}(D_{j}-\overset{―}{D}),$$ (30)

where *W*_*ij*_ represents the spatial weight coefficient relationship between provinces *i* and *j*; $\overset{―}{D}$ represents the mean value of coupling coordination degrees across provinces; ${\text{S}}^{2}$ represents the variance; *D*_*i*_ and *D*_*j*_ represent the coupling coordination indices of provinces *i* and *j* in geographical space. The Moran’s Index value ranges from [-1,1].

### 2.8 Kernel density estimation

Kernel density estimation is a non-parametric method primarily used to describe dynamic data distribution [27]. This method is commonly used to study imbalanced data distribution and can characterize overall distribution features, temporal changes, extensibility, and polarization trends of sample data.

The basic form of kernel density estimation for describing data distribution characteristics is:

$$f(x)=\frac{1}{nh}\sum_{i=1}^{n}k(\frac{x-X_{i}}{h}),$$ (31)

Using the Gaussian kernel function:

$$k(u)=\frac{1}{\sqrt{2\pi }}\exp(-\frac{u^{2}}{2}),$$ (32)

$$u=\frac{x-X_{i}}{h},$$ (33)

where *n* is the sample size; *h* is the bandwidth parameter determining the smoothness of the estimation, with this study using the Silverman criterion to determine the optimal bandwidth parameter; *x* is the point to be estimated; *X*_*i*_ is the observation sequence; k(·) is the kernel density function; *u* is the standardized distance measure.

## 3 Empirical analysis and discussion

### 3.1 Data sources and variable selection

#### 3.1.1 Research sample and data sources.

This study examines 31 provincial-level administrative regions in mainland China (excluding Hong Kong, Macao, and Taiwan) from 2010–2022, providing comprehensive coverage for analyzing coordination patterns across diverse development contexts. Data sources include the China Statistical Yearbook, China Rural Statistical Yearbook, China Ecological Environment Statistical Report, and provincial statistical yearbooks. Missing data points were addressed through interpolation using moving averages to ensure analytical continuity.

The regional classification follows coordination theory’s emphasis on institutional and structural differences that affect dependency management capabilities. The eastern region serves as the pioneer zone for agricultural technological innovation and green transformation, characterized by advanced institutional frameworks and strong coordination mechanisms that enable effective management of resource and outcome dependencies. The central region represents China’s primary grain-producing area where resource and outcome dependencies are most pronounced, playing a crucial role in national food security through intensive agricultural production systems. The western region faces structural development constraints due to resource limitations and institutional capacity restrictions that hinder effective dependency management, but possesses latent comparative advantages in specialty agriculture and ecological agriculture that remain underutilized due to inadequate coordination mechanisms. This classification considers heterogeneity in economic development levels, resource endowments, and agricultural conditions, providing a theoretical foundation for analyzing regional coordination patterns and formulating differentiated agricultural policies. Table 1 presents the detailed provincial composition of each region.

**Table 1:** Regional classification of provincial administrative units.

Region	Number	Provinces
Eastern Region	11	Beijing, Tianjin, Hebei, Liaoning, Shanghai, Jiangsu,
		Zhejiang, Fujian, Shandong, Guangdong, Hainan
Central Region	8	Shanxi, Jilin, Heilongjiang, Anhui, Jiangxi,
		Henan, Hubei, Hunan
Western Region	12	Inner Mongolia, Guangxi, Chongqing, Sichuan, Guizhou,
		Yunnan, Tibet, Shaanxi, Gansu, Qinghai, Ningxia, Xinjiang

Note: Regional classification based on coordination theory principles and institutional capacity for dependency management.

#### 3.1.2 Variable construction and measurement methods.

(1) Agricultural Green Development Efficiency Measurement

Based on the super-efficiency SBM-DEA model, this study constructs a comprehensive evaluation system encompassing inputs, desirable outputs, and undesirable outputs (Table 2). Input indicators capture four resource categories: energy consumption (agricultural diesel, rural electricity), labor resources (rural population, labor force level), capital assets (total mechanical power, sown area), and material inputs (pesticides, fertilizers, plastic film, agricultural water, irrigated area). Desirable outputs reflect green development achievements through certified green food products, certified enterprises, and standardized production base areas, while undesirable outputs measure environmental costs via agricultural COD, carbon emissions, and ammonia nitrogen. This framework enables assessment of how effectively regions achieve agricultural productivity while managing environmental trade-offs.

**Table 2:** Agricultural green development efficiency evaluation indicator system.

Category	Primary Indicator	Secondary Indicator	Unit
Input	Energy Consumption	Agricultural diesel use	10,000 tons
		Rural electricity consumption	100 million kWh
	Labor Input	Rural population	10,000 people
		Labor force level	-
	Capital Input	Total agricultural machinery power	10,000 kW
		Crop sown area	1,000 hectares
	Resource Input	Pesticide usage	10,000 tons
		Chemical fertilizer application	10,000 tons
		Plastic film usage	tons
		Agricultural water consumption	$100\;{\text{million m}}_{3}$
		Effective irrigation area	1,000 hectares
Desirable Output	Green Development Indicators	Number of certified green food products	units
		Number of certified green food enterprises	units
		Green food raw material standardized production base area	10,000 mu
Undesirable Output	Environmental Pollution	Agricultural COD emissions	tons
		Agricultural carbon emissions	tons
		Total ammonia nitrogen emissions	tons

Note: “-” indicates dimensionless indicator.

(2) Food Security Level Measurement

According to food security risk management research [28,29], we establish a comprehensive evaluation model based on the resistance-recovery capacity framework. Studies by [30,31] demonstrate that resistance capacity reflects systems’ ability to prevent and withstand risks, while recovery capacity embodies adaptive responses to disruptions. This two-dimensional approach recognizes that sustainable food security requires both proactive risk prevention and reactive adaptation mechanisms, advancing beyond traditional single-dimensional evaluations.

The entropy TOPSIS method operationalizes this framework through 16 carefully selected indicators (Table 3). Rather than simply measuring production capacity, resistance indicators capture the system’s robustness through economic foundation (agricultural GDP share), resource adequacy (per capita grain possession), risk exposure (disaster-affected areas), and response infrastructure (irrigation systems, transportation networks, meteorological monitoring) [30,31]. Recovery indicators assess adaptive capacity through economic resilience (rural income and consumption levels), social protection (poverty incidence, health services), production adaptability (labor productivity, research investment), and environmental sustainability (drainage capacity, soil conservation) [32,33]. This design enables comprehensive assessment of both immediate stability and long-term adaptive potential, providing dynamic monitoring capabilities for regional food security performance.

**Table 3:** Food security evaluation indicator system.

Primary Indicator	Secondary Indicator	Explanation	Unit	Attribute
Resistance Capacity	Agricultural economic scale	Primary industry added value as proportion of regional GDP	-	+
	Per capita grain possession	Total grain output/Rural total population	kg/person	+
	Crop disaster situation	Crop failure area due to natural disasters	1,000 hectares	-
	Environmental changes	Number of sudden environmental incidents	times	-
	Effective irrigation rate	Effective irrigation area	1,000 hectares	+
	Mechanization level	Total agricultural machinery power	10,000 kW	+
	Road network accessibility	Village road length	km	+
	Meteorological capability	Number of agricultural meteorological observation stations	units	+
Recovery Capacity	Actual income level	Actual per capita net income of rural residents	yuan	+
	Actual consumption level	Per capita consumption expenditure of rural residents	yuan	+
	Rural poverty incidence	Number of rural residents receiving minimum living allowance	-	-
	Agricultural labor productivity	Primary industry added value/Rural total population	100 million yuan/10,000 people	+
	Rural drainage	Cumulative drainage area	1,000 hectares	+
	Public health service	Rural doctors and health workers	persons	+
	Soil erosion control rate	Soil erosion control area/Total crop sown area	-	+
	Agricultural research situation	Agricultural R&D investment intensity	100 million yuan	+

Note: “+” indicates a positive indicator, “-” indicates a negative indicator.

### 3.2 Green agriculture and food security development analysis

#### 3.2.1 Results of agricultural green development efficiency.

China’s agricultural green development efficiency from 2010–2022 reveals substantial regional disparities, with national average efficiency at 0.43 and provincial values ranging from 0.09 to 1.09 (Table 4). The tenfold difference between highest-performing Heilongjiang (1.02) and lowest-performing Henan (0.17) reflects fundamental variations in resource endowments, technological capabilities, and policy implementation effectiveness across regions. Nearly one-third of provinces have efficiency values below 0.30, indicating significant room for improvement in national agricultural green development. The efficiency distribution exhibits a distinctive bimodal pattern: high-efficiency provinces (0.8–1.1 range, 12.9%) demonstrate successful integration of technological innovation and supportive policy frameworks, while the dominant cluster (0.2–0.4 range, 63.4%) represents regions undergoing gradual transformation from quantity-focused to quality-oriented development.

**Table 4:** Agricultural green development efficiency values 2010–2022.

Region	Province	2010	2015	2018	2019	2020	2021	2022	Mean	Rank
Eastern Region	Beijing	0.47	0.49	1.01	1.00	0.66	0.59	0.55	0.76	4
	Fujian	0.25	0.26	0.49	0.32	0.31	0.32	0.32	0.35	16
	Guangdong	0.16	0.15	0.26	0.17	0.17	0.18	0.18	0.19	28
	Hainan	0.32	0.31	0.38	0.35	0.34	0.33	0.33	0.35	17
	Hebei	0.13	0.12	0.26	0.16	0.16	0.17	0.20	0.19	30
	Jiangsu	0.30	0.27	1.03	0.54	0.40	0.40	0.39	0.55	7
	Shandong	0.22	0.19	1.05	0.35	0.32	0.33	0.33	0.48	9
	Shanghai	0.52	0.44	0.85	1.06	1.06	0.86	0.78	0.92	2
	Tianjin	0.42	0.36	0.52	0.42	0.44	0.43	0.46	0.45	10
	Zhejiang	0.28	0.27	1.05	0.38	0.46	0.44	0.42	0.55	8
	Liaoning	0.24	0.25	0.56	0.28	0.26	0.27	0.28	0.33	21
Central Region	Anhui	0.25	0.21	1.09	0.44	0.48	0.42	0.37	0.56	6
	Henan	0.10	0.10	0.20	0.16	0.18	0.16	0.14	0.17	31
	Hubei	0.23	0.22	0.53	0.29	0.30	0.29	0.28	0.34	20
	Hunan	0.22	0.20	0.47	0.34	0.37	0.33	0.30	0.36	15
	Jiangxi	0.23	0.25	0.41	0.29	0.36	0.33	0.30	0.34	18
	Shanxi	0.21	0.18	0.30	0.33	0.38	0.33	0.29	0.32	22
	Heilongjiang	1.03	1.01	1.05	1.01	1.01	1.01	1.01	1.02	1
	Jilin	0.24	0.21	0.49	0.28	0.29	0.28	0.27	0.32	23
Western Region	Gansu	0.27	0.24	0.63	0.35	0.37	0.36	0.34	0.41	13
	Guangxi	0.15	0.14	0.20	0.17	0.22	0.19	0.18	0.19	29
	Guizhou	0.22	0.20	0.26	0.28	0.26	0.24	0.23	0.25	27
	Inner Mongolia	0.31	0.29	0.48	0.39	0.37	0.34	0.32	0.38	14
	Ningxia	0.35	0.38	0.52	0.38	0.40	0.40	0.40	0.42	12
	Qinghai	0.44	0.44	1.01	0.48	0.53	0.52	0.50	0.61	5
	Shaanxi	0.21	0.19	0.32	0.23	0.26	0.25	0.24	0.26	25
	Sichuan	0.24	0.23	0.46	0.31	0.27	0.27	0.26	0.31	24
	Tibet	0.42	0.41	0.44	0.43	0.42	0.42	0.42	0.43	11
	Xinjiang	0.23	0.19	0.31	0.24	0.26	0.24	0.23	0.25	26
	Yunnan	0.20	0.16	0.49	0.39	0.28	0.27	0.26	0.34	19
	Chongqing	0.39	0.30	1.06	0.81	1.01	0.72	0.63	0.84	3
Mean		0.30	0.28	0.59	0.41	0.41	0.38	0.36	0.43	-

Note: The mean column represents the average agricultural green development efficiency of each province in recent five years, and the ranking is sorted from high to low according to the mean value. Data for the periods 2011–2014 and 2016–2017 are not shown in the table for brevity, but were included in the analysis.

Agricultural green development efficiency evolution reflects China’s systematic agricultural policy progression across four distinct periods. The stable foundation period (2010–2016) maintained efficiency levels between 0.30–0.45, characterized by gradual awareness building and initial policy exploration under traditional agricultural development models. The rapid improvement period (2017–2018) witnessed dramatic efficiency surge to 0.59, driven by the convergence of agricultural supply-side structural reforms (initiated in 2015) reaching maturity and the Rural Revitalization Strategy launch in 2017, which together provided institutional frameworks for coordinated development. The framework completion period (2018–2019) saw efficiency peak as the National Green Development Plan for Agriculture (2018–2020) established comprehensive coordination targets and systematic implementation mechanisms, creating policy synergy effects. The strict implementation period (2019–2022) experienced efficiency stabilization around 0.40 as regions adapted to stringent environmental regulations including fertilizer and pesticide reduction targets implemented from 2019, demonstrating the coordination challenges inherent in balancing green transformation with food security objectives under stricter policy constraints.

Heilongjiang Province exemplifies successful green development through a unique combination of natural endowments and institutional innovation. The province maintains exceptional efficiency scores above 1.00 throughout the study period by leveraging three key coordination mechanisms: First, natural advantage integration – utilizing the nation’s largest contiguous black soil area (56% of national black soil zones) combined with land consolidation to achieve an average farm size of 15.6 hectares (21 times the national average), enabling efficient large-scale mechanized operations. Second, technology-scale synergy – establishing a 69.2% agricultural technology contribution rate through innovative ’industry-university-research’ integration models with 23 modern agricultural technology systems, creating a virtuous cycle of ’technological innovation → scale effects → reinvestment → technological upgrading.’ Third, policy-market coordination – developing integrated policy frameworks combining green finance, agricultural insurance, and futures markets, while achieving 20–30% premium pricing for green products through brand building, thus aligning environmental benefits with economic incentives. This model demonstrates how regions can achieve high efficiency by leveraging natural advantages while investing in modern agricultural infrastructure. Eastern coastal regions leverage technological innovation and market integration, while central regions face trade-offs between intensive grain production requirements and environmental constraints, and western regions encounter structural barriers, including fragmented land holdings, limited infrastructure, and challenging natural conditions.

#### 3.2.2 Comprehensive evaluation results of food security.

China’s food security level remained relatively stable but suboptimal during 2010–2022, with national average scores maintaining at 0.26 and fluctuating between 0.25 and 0.30 (Table 5). Even the highest-performing province achieved only 0.64, while nearly one-third of provinces scored below 0.20, indicating substantial improvement potential nationwide. This overall performance reflects the ongoing challenges of balancing intensive agricultural production with sustainable development objectives under resource and environmental constraints.

**Table 5:** Comprehensive measurement values of food security level from 2010 to 2022.

Region	Province	2010	2015	2018	2019	2020	2021	2022	Mean	Rank
Eastern Region	Beijing	0.33	0.32	0.32	0.34	0.33	0.32	0.31	0.32	8
	Fujian	0.22	0.23	0.24	0.24	0.22	0.25	0.27	0.24	13
	Guangdong	0.41	0.40	0.41	0.41	0.42	0.45	0.47	0.43	4
	Hainan	0.17	0.16	0.18	0.18	0.14	0.16	0.17	0.17	23
	Hebei	0.37	0.35	0.32	0.32	0.32	0.32	0.34	0.32	9
	Jiangsu	0.65	0.64	0.65	0.63	0.63	0.64	0.65	0.64	1
	Shandong	0.59	0.59	0.52	0.49	0.49	0.51	0.54	0.51	2
	Shanghai	0.25	0.21	0.20	0.20	0.19	0.20	0.22	0.21	20
	Tianjin	0.19	0.19	0.17	0.17	0.14	0.15	0.15	0.16	25
	Zhejiang	0.29	0.26	0.24	0.23	0.22	0.23	0.26	0.24	14
	Liaoning	0.29	0.23	0.21	0.20	0.19	0.20	0.21	0.20	18
Central Region	Anhui	0.37	0.37	0.36	0.35	0.36	0.37	0.40	0.37	6
	Henan	0.49	0.47	0.44	0.44	0.45	0.46	0.49	0.46	3
	Hubei	0.28	0.28	0.28	0.27	0.27	0.27	0.29	0.28	12
	Hunan	0.24	0.23	0.23	0.22	0.23	0.23	0.23	0.23	15
	Jiangxi	0.19	0.18	0.18	0.18	0.18	0.19	0.20	0.21	18
	Shanxi	0.19	0.17	0.15	0.14	0.14	0.14	0.16	0.15	27
	Heilongjiang	0.43	0.44	0.41	0.41	0.41	0.41	0.44	0.42	5
	Jilin	0.24	0.22	0.19	0.20	0.19	0.20	0.21	0.20	20
Western Region	Gansu	0.21	0.17	0.15	0.16	0.15	0.15	0.17	0.15	26
	Guangxi	0.34	0.30	0.28	0.30	0.27	0.30	0.31	0.29	10
	Guizhou	0.13	0.15	0.16	0.16	0.15	0.16	0.16	0.16	24
	Inner Mongolia	0.24	0.22	0.21	0.21	0.20	0.22	0.23	0.21	17
	Ningxia	0.17	0.14	0.12	0.13	0.11	0.12	0.13	0.12	31
	Qinghai	0.17	0.13	0.13	0.14	0.13	0.14	0.16	0.14	28
	Shaanxi	0.38	0.35	0.33	0.32	0.34	0.36	0.37	0.34	7
	Sichuan	0.28	0.27	0.28	0.27	0.29	0.30	0.30	0.29	11
	Tibet	0.10	0.15	0.11	0.14	0.12	0.14	0.16	0.13	29
	Xinjiang	0.21	0.22	0.21	0.21	0.20	0.24	0.25	0.22	16
	Yunnan	0.17	0.17	0.18	0.19	0.18	0.18	0.19	0.18	22
	Chongqing	0.14	0.13	0.14	0.14	0.12	0.13	0.14	0.13	30
Mean		0.28	0.27	0.28	0.26	0.25	0.26	0.28	0.26	-

Note: The mean column represents the average food security level of each province in recent five years, and the ranking is sorted from high to low according to the mean value. Data for the periods 2011–2014 and 2016–2017 are not shown in the table for brevity, but were included in the analysis.

The period 2018–2020 witnessed a significant downturn, with national levels declining from 0.28 to 0.25 due to three overlapping pressures. Frequent extreme weather events increased average annual crop disaster areas by 15% compared to previous years, testing the system’s resistance capacity. Accelerated industrialization and urbanization resulted in approximately 1.2% annual reduction in quality farmland, constraining production foundations. Additionally, insufficient agricultural infrastructure investment, with annual growth rates below 5%, weakened the system’s adaptive capacity. These factors collectively exposed vulnerabilities in China’s food security framework during a critical development transition period. The post-2020 recovery demonstrates remarkable system resilience, with national levels rising from 0.25 to 0.28 (a 12% increase) through targeted policy interventions. The High-Standard Farmland Construction Program and enhanced grain reserve management strengthened both resistance and recovery capacities. Jiangsu Province exemplifies successful integration, achieving a national-leading score of 0.64 through comprehensive agricultural modernization that combines advanced production technologies with robust risk management systems. Conversely, provinces like Tibet (0.13) illustrate the persistent challenges faced by regions with harsh environmental conditions and limited agricultural diversification options, highlighting the need for differentiated development strategies.

### 3.3 Analysis of coupling coordination degree results

#### 3.3.1 Temporal evolution characteristics.

The coupling coordination between agricultural green development efficiency and food security exhibits three distinct evolutionary phases from 2010–2022 (Table 6), which closely correspond to China’s agricultural policy evolution and coordination theory’s understanding of how complex systems develop coordinated relationships through dependency management.

**Table 6:** Coupling coordination degree between agricultural green development efficiency and food security from 2010 to 2022.

Region	Province	2010	2015	2018	2019	2020	2021	2022	Mean	Rank
Eastern Region	Beijing	0.63	0.63	0.76	0.76	0.68	0.66	0.64	0.70	3
	Fujian	0.49	0.49	0.58	0.52	0.51	0.53	0.54	0.54	12
	Guangdong	0.51	0.50	0.57	0.51	0.52	0.53	0.54	0.53	15
	Hainan	0.48	0.47	0.51	0.50	0.47	0.48	0.49	0.49	25
	Hebei	0.47	0.45	0.54	0.48	0.47	0.49	0.51	0.50	23
	Jiangsu	0.67	0.64	0.90	0.76	0.71	0.71	0.71	0.76	2
	Shandong	0.60	0.58	0.86	0.64	0.63	0.64	0.65	0.69	4
	Shanghai	0.60	0.55	0.64	0.68	0.67	0.65	0.64	0.66	6
	Tianjin	0.53	0.51	0.55	0.51	0.50	0.50	0.52	0.51	18
	Zhejiang	0.53	0.51	0.71	0.54	0.56	0.57	0.58	0.59	7
Central Region	Liaoning	0.51	0.49	0.59	0.49	0.47	0.49	0.49	0.51	19
	Anhui	0.55	0.53	0.79	0.63	0.64	0.63	0.62	0.66	5
	Henan	0.47	0.47	0.54	0.52	0.53	0.52	0.51	0.52	17
	Hubei	0.50	0.50	0.62	0.53	0.53	0.53	0.53	0.55	9
	Hunan	0.48	0.46	0.57	0.52	0.54	0.52	0.51	0.53	13
	Jiangxi	0.46	0.46	0.52	0.48	0.50	0.50	0.49	0.50	21
	Shanxi	0.45	0.42	0.46	0.46	0.48	0.46	0.46	0.47	30
	Heilongjiang	0.82	0.81	0.81	0.80	0.80	0.80	0.81	0.81	1
	Jilin	0.49	0.46	0.55	0.48	0.49	0.49	0.49	0.50	20
Western Region	Gansu	0.49	0.45	0.55	0.48	0.48	0.48	0.49	0.50	22
	Guangxi	0.48	0.45	0.49	0.47	0.49	0.49	0.49	0.48	28
	Guizhou	0.41	0.41	0.45	0.46	0.44	0.44	0.44	0.45	31
	Inner Mongolia	0.52	0.50	0.56	0.53	0.52	0.52	0.52	0.53	16
	Ningxia	0.50	0.48	0.50	0.47	0.46	0.47	0.48	0.47	29
	Qinghai	0.52	0.49	0.60	0.51	0.51	0.52	0.53	0.53	14
	Shaanxi	0.53	0.51	0.57	0.52	0.54	0.55	0.55	0.55	11
	Sichuan	0.51	0.50	0.60	0.54	0.53	0.53	0.53	0.55	10
	Tibet	0.45	0.50	0.47	0.49	0.48	0.49	0.51	0.49	26
	Xinjiang	0.46	0.45	0.51	0.47	0.48	0.49	0.49	0.49	27
	Yunnan	0.43	0.41	0.54	0.52	0.47	0.47	0.47	0.50	24
	Chongqing	0.48	0.44	0.62	0.58	0.59	0.55	0.54	0.58	8
Mean		0.52	0.50	0.60	0.55	0.54	0.54	0.54	0.55	-

Note: The mean column represents the average coupling coordination degree of each province in recent five years, and the ranking is sorted from high to low according to the mean value. Data for the periods 2011–2014 and 2016–2017 are not shown in the table for brevity, but were included in the analysis.

The stable development period (2010–2016) represents the dependency recognition phase, where coordination levels maintained between 0.49–0.52, reflecting the gradual adaptation of traditional agricultural systems to emerging environmental constraints. During this phase, regions primarily focused on incremental improvements within existing institutional frameworks, as they began to recognize the interdependencies between green development and food security systems. The agricultural supply-side structural reforms initiated in 2015 provided the conceptual foundation for systematic coordination, though their full effects had not yet materialized, resulting in steady but limited coordination progress. The rapid improvement period (2017–2018) corresponds to the mechanism development phase, witnessing a dramatic coordination breakthrough with levels surging to 0.60. This transformation resulted from policy convergence effects: the maturation of agricultural supply-side structural reforms (2015) created institutional readiness, while the Rural Revitalization Strategy launch in 2017 provided comprehensive frameworks for coordinated development. The National Green Development Plan for Agriculture (2018–2020) further established explicit coordination targets, creating synergistic policy momentum that enabled regions to simultaneously pursue efficiency improvements and food security enhancement, demonstrating how coordinated policy frameworks can overcome traditional trade-offs between environmental and production objectives. The stable adjustment period (2019–2022) reflects the adaptive management phase, experiencing coordination stabilization around 0.54 as regions integrated stricter environmental regulations with productivity requirements. The implementation of fertilizer and pesticide reduction targets in 2019, while environmentally beneficial, initially disrupted established production patterns and required institutional learning to maintain coordination levels. This adjustment phase reveals that sustaining high-level coordination requires continuous institutional adaptation rather than one-time policy interventions.

#### 3.3.2 Statistical validation of temporal trends.

**Fig 2:**
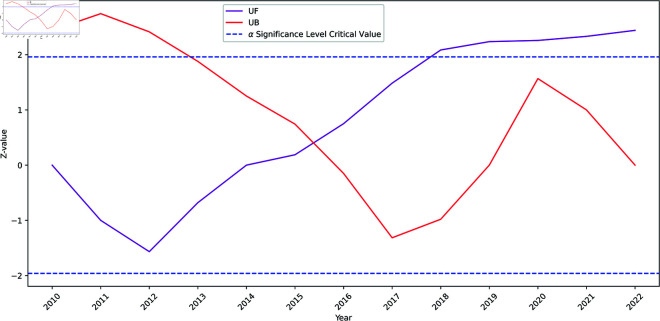
Mann-Kendall trend test results for coupling coordination development. The figure displays the Mann-Kendall statistical analysis of coupling coordination between agricultural green development efficiency and food security from 2010–2022. The upper and lower horizontal dashed lines represent the critical values at α=0.05 significance level (±1.96). The purple line (UF) represents the forward sequence standard normal distribution, while the red line (UB) denotes the backward sequence standard normal distribution. The intersection of UF and UB curves around 2016–2017 indicates a potential structural change point within the critical confidence interval.

To establish the statistical significance of observed temporal evolution trends, this study conducts Mann-Kendall trend analysis, which validates the proposed three-phase development pattern and confirms that most provinces experienced significant coordination improvements, with regional variations reflecting different development trajectories and policy implementation effectiveness (Fig 2 and Table 7). Fig 2 reveals that China’s coupling coordination demonstrates a statistically significant upward trend during 2010–2022, with the UF curve exceeding the significance threshold from approximately 2018 onwards. The national Mann-Kendall test yields a Z-statistic of 2.379 with a p-value of 0.017 (*p* < 0.05), confirming the statistical significance of the increasing trend. The Kendall’s τ coefficient of 0.513 indicates a moderate positive trend strength, while Sen’s slope estimator reveals an annual growth rate of 0.48%. Crucially, the mutation point detection validates our proposed phase transitions. The intersection of UF and UB curves occurs around 2016–2017 within the critical confidence interval (±1.96), representing a statistically significant structural change point. This empirical evidence strongly supports the transition from the stable development period to the rapid improvement period, coinciding with agricultural supply-side structural reforms and providing a statistical foundation for our three-stage development framework. From a coordination theory perspective, this structural change point indicates a fundamental shift in dependency management mechanisms, where policy convergence enabled more effective coordination between environmental sustainability and food security objectives.

**Table 7:** Mann-Kendall trend test results for coupling coordination degree 2010–2022.

Region	Province	P-value	Z-value	Kendall’s Tau	Sen’s Slope (%)
Eastern Region	Beijing	0.024*	2.257	0.487	0.50
	Fujian	0.004**	2.867	0.615	0.60
	Guangdong	0.004**	2.867	0.615	0.30
	Hainan	0.246	1.159	0.256	0.10
	Hebei	0.161	1.403	0.308	0.30
	Jiangsu	0.024*	2.257	0.487	0.70
	Shandong	0.006**	2.745	0.590	0.80
	Shanghai	0.017*	2.379	0.513	1.00
	Tianjin	0.583	-0.549	–0.128	–0.10
	Zhejiang	0.004**	2.867	0.615	0.70
	Liaoning	0.951	–0.061	–0.026	0.00
Central Region	Anhui	0.017*	2.379	0.513	1.30
	Henan	0.024*	2.257	0.487	0.60
	Hubei	0.002**	3.111	0.667	0.50
	Hunan	0.017*	2.379	0.513	0.70
	Jiangxi	0.004**	2.867	0.615	0.50
	Shanxi	0.044*	2.013	0.436	0.30
	Heilongjiang	0.428	0.793	0.179	0.30
	Jilin	0.100	1.647	0.359	0.30
Western Region	Gansu	0.428	0.793	0.179	0.20
	Guangxi	0.161	1.403	0.308	0.10
	Guizhou	0.001**	3.233	0.692	0.40
	Inner Mongolia	0.077	1.769	0.385	0.30
	Ningxia	0.300	–1.037	–0.231	–0.20
	Qinghai	0.300	1.037	0.231	0.10
	Shaanxi	0.127	1.525	0.333	0.20
	Sichuan	0.024*	2.257	0.487	0.40
	Tibet	0.161	1.403	0.308	0.30
	Xinjiang	0.009**	2.623	0.564	0.40
	Yunnan	0.033*	2.135	0.462	0.80
	Chongqing	0.044*	2.013	0.436	1.00
Mean		0.017*	2.379	0.513	0.48

Note: * indicates significance at *p* < 0.05 level; ** indicates significance at *p* < 0.01 level. Sen’s slope represents the annual change rate in coupling coordination degree.

Provincial-level analysis further confirms the widespread nature of coordination improvements across different regional contexts, with results presented in Table 7. Eastern regions demonstrate the strongest statistical performance, with 7 out of 11 provinces showing significant trends, central regions display robust improvement with 6 out of 8 provinces achieving significance, while western regions present more heterogeneous results reflecting varied development trajectories and policy implementation effectiveness. These regional variations in statistical significance reflect differential capacities for managing resource and outcome dependencies, with eastern regions demonstrating more mature coordination mechanisms and western regions facing structural constraints in dependency management.

#### 3.3.3 Spatial distribution patterns.

**Fig 3:**
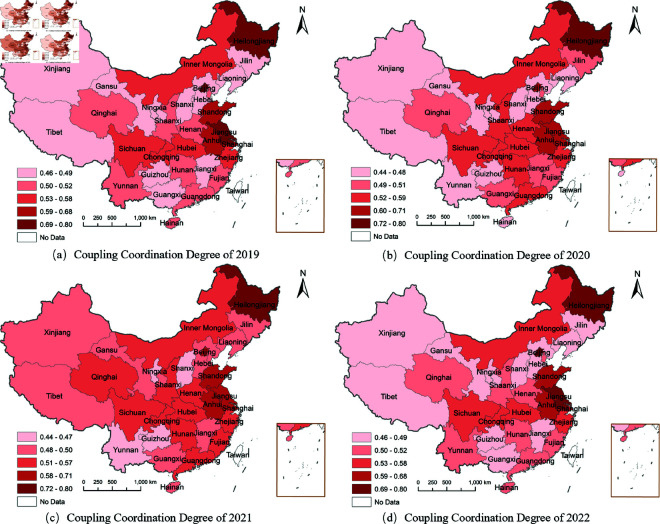
The coupling coordination degree distribution map of China from 2019 to 2022. The maps illustrate the spatial evolution of coupling coordination between agricultural green development efficiency and food security across Chinese provinces. (a) Coupling Coordination Degree of 2019. (b) Coupling Coordination Degree of 2020. (c) Coupling Coordination Degree of 2021. (d) Coupling Coordination Degree of 2022.

To visualize the spatial distribution patterns of coupling coordination degree, Fig 3 presents the distribution maps from 2019 to 2022. These maps clearly demonstrate the evolution of regional development patterns and the formation of high-value clusters across China’s provinces. Heilongjiang Province stands out with exceptional performance, maintaining an average coupling coordination degree of 0.81 and consistently staying within the high-level interval throughout the study period. This success stems from its large-scale mechanized agriculture model supported by substantial government investment in modern agricultural infrastructure and the National Modern Agriculture Demonstration Zone designation, which provided preferential policies for equipment subsidies and technological upgrades.

The eastern coastal regions have formed high-level development clusters leveraging their economic advantages and innovation capacity. Jiangsu (0.76), Beijing (0.70), and Shandong (0.69) achieved significant coordination improvements after 2017, with Jiangsu demonstrating remarkable progress from 0.67 in 2010 to 0.90 in 2018. This dramatic improvement resulted from the province’s comprehensive agricultural modernization program, which invested over 50 billion yuan in high-standard farmland construction, smart agriculture technologies, and green production systems. The success demonstrates how targeted investment in agricultural infrastructure and technology can rapidly enhance coordination between efficiency and security objectives.

The maps reveal a pronounced spatial gradient from eastern coastal regions to western inland areas. Eastern provinces consistently maintain coordination levels above 0.65, benefiting from higher agricultural R&D investment (averaging 3.2% of agricultural GDP compared to 1.8% nationally), better transportation infrastructure that reduces post-harvest losses by 15–20%, and more diversified agricultural industrial chains that provide farmers with premium market access. Western regions predominantly remain below 0.50, facing structural development constraints including limited infrastructure investment, fragmented land holdings averaging 0.6 hectares per household compared to 2.1 hectares in northeastern regions, and restricted access to advanced agricultural technologies due to lower education levels and limited extension services. However, these regions possess latent comparative advantages in ecological agriculture and specialty products, with unique natural endowments for organic farming and high-altitude agriculture that remain underutilized due to inadequate coordination mechanisms and insufficient market integration.

The persistent spatial disparities observed under unified national policies highlight the limitations of one-size-fits-all approaches to agricultural development. Despite implementing consistent policy frameworks across all provinces, regional coordination outcomes vary dramatically, with eastern regions achieving coordination levels nearly twice those of western areas. This divergence suggests that effective policy design must account for regional heterogeneity in resource endowments, development foundations, and institutional capacity. Future agricultural policy should adopt differentiated strategies that align intervention types and intensity with specific regional characteristics and development stages. Eastern regions may benefit from policies emphasizing technological innovation and industrial upgrading, while western regions require fundamental infrastructure investment and capacity-building support to establish the prerequisites for effective coordination between green development and food security objectives.

### 3.4 Regional difference analysis

This section employs Dagum’s Gini coefficient decomposition method to systematically examine the regional differentiation characteristics of coupling coordination between agricultural green development efficiency and food security from 2010 to 2022. By decomposing overall inequality into three dimensions – within-region differences, between-region differences, and transvariation intensity – the study reveals the structural characteristics and evolution patterns of regional differences (Table 8). The overall Gini coefficient peaked at 0.100 in 2018 before declining to 0.072 in 2022. The significant rise in the Gini coefficient during 2017–2018 coincided with the deepening period of agricultural supply-side reform, when regional differences in policy implementation, resource allocation, and technology application intensified regional divergence. After 2020, as the nation strengthened coordinated promotion of food security and agricultural green development, regional differences showed a convergence trend.

**Table 8:** Dagum gini coefficient and its decomposition results.

Year	Overall	Within-Region			Between-Region			Contribution Rate (%)		
		East	Cent	West	E-C	E-W	C-W	Within	Between	Transvariation
2010	0.073	0.064	0.093	0.043	0.091	0.074	0.091	28.54	40.78	30.69
2011	0.065	0.057	0.073	0.043	0.082	0.067	0.082	28.59	41.32	30.09
2012	0.065	0.060	0.069	0.044	0.078	0.070	0.078	29.07	43.67	27.26
2013	0.067	0.064	0.072	0.048	0.080	0.072	0.080	29.89	38.70	31.41
2014	0.074	0.066	0.090	0.048	0.092	0.074	0.092	29.23	37.20	33.57
2015	0.073	0.063	0.097	0.040	0.092	0.072	0.092	28.13	40.86	31.01
2016	0.076	0.065	0.100	0.037	0.096	0.079	0.096	27.23	45.21	27.55
2017	0.092	0.094	0.100	0.045	0.102	0.106	0.102	27.96	49.99	22.05
2018	0.100	0.106	0.104	0.056	0.111	0.113	0.111	29.24	45.52	25.25
2019	0.082	0.096	0.092	0.039	0.099	0.089	0.099	29.76	40.89	29.35
2020	0.079	0.086	0.088	0.042	0.092	0.082	0.092	29.59	36.19	34.23
2021	0.074	0.078	0.089	0.037	0.088	0.078	0.088	29.10	39.08	31.82
2022	0.072	0.069	0.092	0.034	0.088	0.077	0.088	28.04	43.61	28.35

Note: Within-Region refers to Gini coefficients within Eastern (East), Central (Cent), and Western (West) regions. Between-Region shows coefficients between East-Central (E-C), East-West (E-W), and Central-West (C-W). Contribution Rate shows percentages for Within-Region (Within), Between-Region (Between), and Transvariation Intensity (Transvariation).

Between-region differences reflect a typical “gradient effect”: eastern regions lead agricultural modernization transformation through economic and technological advantages, while central and western regions lag in coordinated development. These kinds of differences consistently dominate inequality sources, contributing 36.19%–49.99% compared to within-region differences (27.23%–29.89%), indicating that inequality stems primarily from systematic disparities rather than random variations.

The persistent dominance of between-region differences reveals that regional development imbalance remains the key constraint on coordination improvement. This gradient effect reflects systematic disparities in development foundations, institutional capacity, and resource endowments that transcend individual provincial variations. Eastern regions like Jiangsu and Zhejiang have achieved high coordination through leveraging economic and technological advantages, establishing comprehensive innovation systems that enable simultaneous efficiency gains and food security enhancement. Central regions including Henan and Hubei face the dual challenge of maintaining national food production responsibilities while meeting environmental sustainability requirements, requiring targeted support for sustainable intensification technologies. Western regions such as Tibet and Xinjiang encounter structural constraints from geographic conditions and limited infrastructure, but demonstrate potential for location-specific development through ecological agriculture and resource-efficient technologies.

Future policy should prioritize differentiated regional strategies that address specific coordination challenges. Eastern regions need policies supporting technology transfer mechanisms and innovation spillover to other regions. Central regions require sustainable intensification support that balances production intensity with environmental protection. Western regions need foundational infrastructure investment and development of comparative advantages in ecological agriculture. The declining inequality trend after 2020 suggests that coordinated regional policies can effectively reduce disparities when designed to leverage regional strengths while addressing specific constraints.

### 3.5 Spatial correlation analysis

This section employs both global and local Moran’s index methods to systematically analyze the spatial association patterns of coordinated development between agricultural green development efficiency and food security from 2010 to 2022, revealing significant spatial autocorrelation characteristics and policy implications for regional coordination enhancement.

The global Moran’s index demonstrates significant spatial autocorrelation with distinct temporal phases that directly correspond to China’s agricultural policy evolution (Table 9). During 2010–2015, the index continuously declined from 0.18 to 0.10, indicating weakening spatial clustering as regions pursued divergent development paths under traditional agricultural modes, with limited inter-regional coordination mechanisms. The period 2016–2018 witnessed rapid strengthening of spatial correlation, with Moran’s index reaching 0.31 (Z = 3.02, *p* < 0.01), driven by the convergence of multiple policy interventions: the maturation effects of agricultural supply-side structural reforms (2015), the comprehensive coordination frameworks established by the Rural Revitalization Strategy (2017), and the systematic implementation guidelines provided by the National Green Development Plan for Agriculture (2018–2020). These policies collectively enhanced factor mobility and technology diffusion across regions, creating stronger spatial interdependence. The subsequent stabilization around 0.18–0.20 (2019–2022) indicates sustained but moderate spatial interdependence, suggesting that while initial policy momentum created lasting regional connectivity effects, the implementation of stricter environmental regulations including fertilizer and pesticide reduction targets from 2019 required regions to focus more on internal adaptation, slightly reducing cross-regional spillover intensity. This “U-shaped” evolution trajectory validates the importance of policy frameworks in facilitating spatial coordination. The strengthening agglomeration effects after 2016 correspond directly to the advancement of regional coordinated development strategies, particularly the implementation of cross-regional collaboration mechanisms that significantly enhanced spatial correlation through institutional alignment and knowledge transfer networks.

**Table 9:** Spatial correlation test of coupling coordination development level.

Year	2010	2011	2012	2013	2014	2015	2016	2017	2018	2019	2020	2021	2022
Moran’s I	0.18	0.18	0.18	0.12	0.12	0.10	0.13	0.26	0.31	0.15	0.17	0.18	0.20
E(I)	–0.03	–0.03	–0.03	–0.03	–0.03	–0.03	–0.03	–0.03	–0.03	–0.03	–0.03	–0.03	–0.03
Sd(I)	0.11	0.11	0.11	0.11	0.11	0.10	0.11	0.11	0.12	0.11	0.11	0.11	0.11
Z	2.04	1.98	1.89	1.35	1.34	1.28	1.58	2.56	3.02	1.64	1.83	1.91	2.10
P-value	0.04*	0.05*	0.06	0.18	0.18	0.20	0.11	0.01**	0.00**	0.10	0.07	0.06	0.04*

Note: * indicates significance at 5% level (*p* < 0.05); ** indicates significance at 1% level (*p* < 0.01). E(I) represents the expected value of Moran’s I under the null hypothesis of no spatial autocorrelation, calculated as −1/(n−1) where n = 31. Sd(I) denotes the standard deviation of Moran’s I.eak The Z-value is calculated as [I−E(I)]/Sd(I) and follows a standard normal distribution under the null hypothesis.

**Fig 4:**
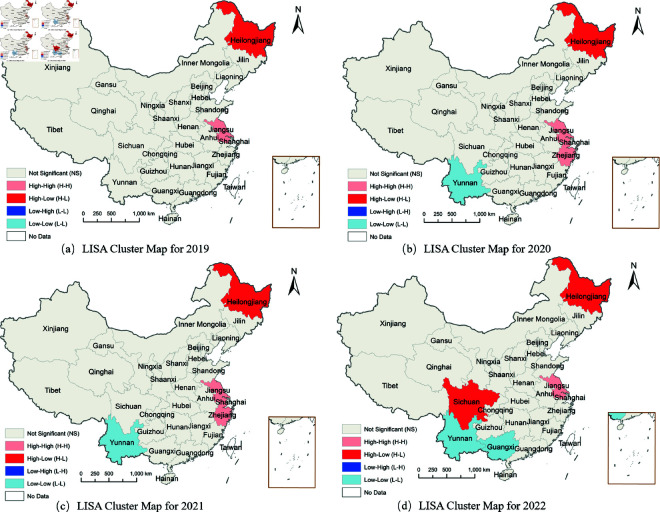
LISA cluster map based on local Moran’s I, 2019–2020. The maps illustrate the spatial distribution of Local Moran’s I clustering for agricultural green development and related factors across Chinese provinces. (a) LISA Cluster Map for 2019. (b) LISA Cluster Map for 2020. (c) LISA Cluster Map for 2021. (d) LISA Cluster Map for 2022.

To further examine local clustering dynamics, LISA cluster maps were generated to identify statistically significant spatial associations in coupling coordination development [34]. Fig 4 analysis reveals distinct spatial regimes that validate our multi-dimensional analytical framework, with each cluster type representing different coordination development mechanisms.

High-high clusters represent coordination innovation cores with strong local spillover effects. Jiangsu Province consistently maintains this status throughout the period with statistical significance at *p* < 0.05 level, demonstrating sustained coordination leadership. Zhejiang Province joins this high-high cluster category in 2020–2021, also achieving statistical significance at *p* < 0.05 level. However, Zhejiang’s absence from the high-high category in 2022 reveals the dynamic and sometimes unstable nature of coordination achievements, suggesting that maintaining high-level coordination requires continuous institutional adaptation rather than one-time policy success. These regions emerge as epicenters of harmonized green agriculture and food security development, exemplifying the successful integration of environmental sustainability and food production.

High-low clusters indicate isolated excellence with untapped spillover potential. Heilongjiang consistently appears in this category throughout 2019–2022, demonstrating the highest statistical significance at *p* < 0.01 level. Sichuan joins this pattern in 2022. This pattern underscores their potential as regional development poles that have achieved high coordination levels but have not yet generated significant spillover effects to neighboring regions, reflecting the spatial interaction limitations identified in our Moran’s index analysis.

Low-low clusters represent coordination development challenges requiring structural interventions. The concerning expansion pattern shows Yunnan consistently appearing in this category from 2020–2021 with statistical significance at *p* < 0.05 level, joined by Guangxi in 2022, also at *p* < 0.05 significance level. These clusters are primarily concentrated in southwestern China, representing regions facing persistent coordination challenges. This spatial concentration of low-coordination areas aligns with our Gini decomposition findings that between-region differences dominate inequality patterns.

The LISA results provide spatial validation for our comprehensive analytical framework. The limited extent of statistically significant clustering (compared to the descriptive patterns in Fig 3) suggests that China’s regional coordination mechanisms, while showing promise, still face challenges in creating robust spatial linkages across provincial boundaries.

### 3.6 Kernel density estimation analysis

**Fig 5:**
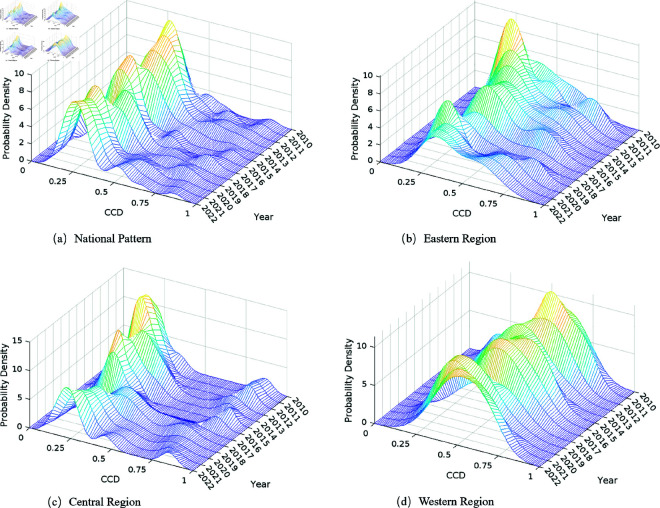
Kernel density distribution map. The three-dimensional kernel density maps show the dynamic evolution of coupling coordination distribution patterns. (a) National Pattern. (b) Eastern Region. (c) Central Region. (d) Western Region.

Kernel density estimation provides dynamic visualization of coupling coordination distribution evolution, revealing regional convergence and divergence trends that inform policy design for balanced development [35]. Fig 5 shows that the national coordination distribution exhibits clear polarization dynamics over time. In 2010, the distribution showed a concentrated single-peak pattern around 0.4–0.5 with high density (8–10), indicating relatively uniform development across regions. By 2022, this evolved into a flattened, multi-modal distribution with lower peak density (4–6) but wider spread, signaling regional divergence rather than convergence. This transformation reveals that uniform national policies have produced differentiated outcomes, with regions following distinct development trajectories based on their initial conditions and adaptive capacities. Different coordination levels across regions indicate that a one-size-fits-all policy approach may be insufficient for achieving balanced development. The flattening of the distribution density implies that achieving coordination improvements has become more differentiated and complex over time.

Regional analysis reveals distinct evolutionary characteristics that reflect different development models and constraints. The eastern region demonstrates “single-peak evolution” with clear path advantages. The density peak migrated rightward from 0.5 to 0.75 interval while intensifying above 8, forming a stable dominant peak in the 0.6–0.8 range with good symmetry. This pattern reflects the region’s superior policy implementation capacity, market system, and resource allocation efficiency, enabling consistent coordination improvements even during transition periods (after 2015).

The central region presents unique “double-peak evolution” characteristics, forming a distinct double-peak pattern that reflects three fundamental differentiation mechanisms. First, resource constraint differentiation – provinces like Henan and Hubei face intense pressure with limited per capita arable land (1.2–1.5 mu) and must maintain 60% of national grain production, creating path dependency on chemical inputs, while provinces like Hunan and Jiangxi possess better land quality and greater transformation flexibility. Second, policy transmission heterogeneity – identical national policies produce divergent outcomes due to differences in fiscal capacity, governance capability, and industrial foundations, with financially stronger provinces like Anhui and Hubei achieving better policy implementation through “policy-funding-technology-outcome” cycles. Third, industrial structure path-locking – “high-peak” provinces achieved earlier agricultural industrialization and scale development, establishing comprehensive agricultural value chains that facilitate green transformation, while “low-valley” provinces remain locked in traditional family farming patterns lacking the industrial foundation for coordinated development.

The western region demonstrates “low-level dispersion” characteristics that remained relatively stable throughout the study period. The density surface is generally flat with maximum density reaching only 5-6, primarily distributed in the low coordination interval of 0.3–0.5. Multiple small peaks reveal significant internal heterogeneity, correlating with the region’s complex geographical environment and differentiated agricultural production conditions. The persistent low development momentum indicates limited effectiveness of existing Chinese government policy support in overcoming structural constraints.

The evolutionary patterns suggest that coordination development exhibits strong regional path dependency, where early institutional investments and development foundations determine long-term trajectories. The eastern region’s consistent improvement reflects cumulative advantages from early coordination capacity building, while the western region’s stable low performance indicates the need for fundamental structural interventions rather than incremental policy adjustments. The central region’s bifurcated pattern suggests that targeted interventions can be effective but require addressing specific regional constraints to prevent development fragmentation.

These results of Kernel Density Estimation Analysis indicate that future policy design should recognize these distinct regional dynamics: supporting the eastern region’s demonstration role and spillover effects; addressing development gaps within the central region through targeted coordination mechanisms; and implementing comprehensive capacity building in the western region that addresses fundamental structural barriers to coordinated development. Simultaneously, a more comprehensive regional coordinated development mechanism should be established to promote efficient factor flow and achieve positive interaction between agricultural green development and food security.

## 4 Conclusions and recommendations

This study examined the coupling coordination relationship between agricultural green development efficiency and food security in China from 2010 to 2022, demonstrating that coordinated advancement of agricultural green development and food security can develop simultaneously. Drawing upon coordination theory, the research reveals that successful coordination emerges through effective management of resource, temporal, and outcome dependencies rather than trade-offs between environmental and production objectives. Provinces such as Heilongjiang and Jiangsu have achieved unity between high productivity and green transformation, maintaining high-level coupling coordination degrees above 0.8 through innovative dependency management approaches. However, this coordination model has not yet been widely adopted nationwide, with over 60% of regions still at relatively low coordination levels, particularly in areas with weak institutional capacity and limited resource endowments.

Following a comprehensive analytical framework, this study integrated efficiency measurement using super-efficiency SBM-DEA model, food security assessment through entropy TOPSIS method, coupling coordination degree analysis, spatial correlation analysis, regional difference decomposition, and kernel density estimation to provide robust empirical evidence for policy design. The research obtained comprehensive scores for agricultural green development efficiency and food security levels across all provinces nationwide, revealing evolution through three distinct phases: stable development, rapid improvement, and stable adjustment periods. The temporal evolution demonstrates structural transitions coinciding with major policy interventions like the Rural Revitalization Strategy, suggesting that coordinated policy frameworks can effectively break traditional trade-offs between environmental protection and food production.

Analysis reveals pronounced regional differentiation with distinct development clusters reflecting fundamental differences in institutional capacity and resource endowments. Three major high-value clusters formed around Beijing-Tianjin-Hebei, the Yangtze River Delta, and Heilongjiang, driven by superior innovation systems, policy support, and natural advantages respectively. Regional stratification persists due to cumulative development advantages: eastern regions achieved stable high-level development through early investments in technological innovation and institutional capacity; central regions experience critical transition periods as they navigate between intensive production pressures and environmental sustainability requirements; western regions face insufficient development momentum constrained by geographical limitations and weaker institutional foundations. Regional difference analysis demonstrates that between-region differences dominate inequality sources, indicating that these disparities are systematic rather than random phenomena rooted in structural development foundations. Regional connectivity has strengthened after policy interventions due to enhanced factor mobility and cross-regional collaboration mechanisms, though agglomeration effects concentrate primarily in developed eastern regions where existing advantages create self-reinforcing development cycles. Distribution pattern analysis identifies divergent trends reflecting path-dependent development trajectories, with eastern regions showing convergence toward high coordination levels through institutional learning, while central regions exhibit concerning bifurcation due to heterogeneous adaptation capabilities, and western regions maintain persistent low-level dispersion constrained by structural barriers to coordination development.

The multi-dimensional spatial analytical framework provides robust and complementary evidence for these regional patterns. Gini coefficient decomposition quantitatively confirms the dominance of between-region differences (36.19%–49.99% versus within-region differences at 27.23%–29.89%), validating the systematic nature of spatial inequality. Moran’s index analysis reveals the spatial interaction mechanisms underlying these patterns, demonstrating strengthening agglomeration effects after 2016 (Moran’s I reaching 0.31) that create and reinforce high-value clusters in eastern regions through spatial spillovers and knowledge transfer. Kernel density estimation captures the evolutionary dynamics of these spatial patterns, showing a transition from concentrated single-peak distribution (2010) to flattened multi-modal patterns (2022), providing empirical evidence for regional divergence rather than convergence. These three analytical approaches converge on a consistent conclusion: China’s coordination development exhibits path-dependent spatial stratification where initial regional advantages create self-reinforcing mechanisms, systematic structural differences persist and intensify over time, and uniform national policies produce differentiated regional outcomes requiring targeted interventions.

Based on empirical typologies, this study proposes targeted policy recommendations addressing both national coordination frameworks and region-specific interventions.

(1) National-level Coordination Framework Comprehensive coordination frameworks should be established to promote collaborative regional development and address pronounced regional development imbalances. These frameworks should facilitate the flow of production factors including technology, personnel, and capital across regions while establishing unified cooperation mechanisms that enable regions to leverage their comparative advantages collectively. Cross-cutting coordination mechanisms should include improved inter-regional policy coordination, enhanced agricultural ecological compensation systems that reward environmental improvements, innovative financial service models including agricultural green insurance and credit support, and unified national monitoring networks for real-time tracking of coordination indicators.

(2) High-coordination Regions For eastern coastal areas and Heilongjiang among other high-coordination regions, policies should emphasize technology transfer leadership and demonstration effects, positioning these areas as key nodes in the national coordination framework. These regions need support for establishing agricultural technology innovation centers, cross-regional knowledge transfer platforms, and international market integration mechanisms that leverage their coordination advantages to drive broader regional development and fulfill their responsibilities as national demonstration zones.

(3) Transitional Coordination Regions For central grain-producing areas and similar transitional coordination regions, policies should address the dual challenge of maintaining food production responsibilities while achieving environmental sustainability within the national food security framework. Specific interventions include sustainable intensification technology support, soil health restoration programs, climate-resilient crop variety development, and integrated pest management systems that enable coordination improvement without compromising food security functions.

(4) Low-coordination Regions For western provinces and other low-coordination regions, policies require comprehensive structural interventions addressing fundamental capacity constraints while integrating these areas into national development networks. Priority measures include infrastructure investment in transportation and irrigation systems, institutional capacity building for agricultural extension services, development of location-specific agricultural systems that leverage ecological advantages, and targeted financial support for farmers transitioning to sustainable practices, all supported by enhanced connectivity to national coordination frameworks.
